# Comparison of minimally invasive transforaminal lumbar interbody fusion (Mis-TLIF) with bilateral decompression via unilateral approach and open-TLIF with bilateral decompression for degenerative lumbar diseases: a retrospective cohort study

**DOI:** 10.1186/s13018-024-04630-1

**Published:** 2024-02-20

**Authors:** Fengzhao Zhu, Dongqing Jia, Yaqing Zhang, Chencheng Feng, Ya Ning, Xue Leng, Yue Zhou, Changqing Li, Bo Huang

**Affiliations:** 1 Department of Orthopedics, Xinqiao Hospital, Army Medical University, Chongqing, China; 2 Department of Blood Transfusion, University-Town Hospital of Chongqing Medical University, Chongqing, China

**Keywords:** Minimally invasive transforaminal lumbar interbody fusion, Bilateral decompression via unilateral approach, Open-TLIF, Degenerative lumbar diseases, Learning curve

## Abstract

**Objective:**

Presently, no study has compared the clinical outcomes of minimally invasive transforaminal lumbar interbody fusion (Mis-TLIF) with bilateral decompression via the unilateral approach (BDUA) and Open-TLIF with bilateral decompression for degenerative lumbar diseases (DLD). We aimed to compare the clinical outcomes of through Mis-TLIF combined with BDUA and Open-TLIF with bilateral decompression for the treatment of DLD, and reported the learning curve of the procedure of MIS-TLIF with BDUA.

**Methods:**

We retrospectively analyzed the prospectively collected data of consecutive DLD patients in the two groups from January 2016 to January 2020.

**Results:**

The operative time (OT) was significantly longer in the Mis-TLIF group (*n* = 113) than in the Open-TLIF group (*n* = 135). The postoperative drainage volume (PDV) and length of stay (LOS) were significantly higher in the Open-TLIF group than in the Mis-TLIF group. Additionally, the complication rate was significantly higher in the Open-TLIF group than in the Mis-TLIF group (14.8% vs. 6.2%, *P* = 0.030), while there was no significant difference in the reoperation and adjacent segment disease rates between the two groups. There were no significant differences in back pain and leg pain Numerical Rating Scale (NRS) scores and Oswestry Disability Index (ODI) between the two groups preoperatively, at discharge, and 2 years postoperatively. Patients in both groups showed significant improvements in NRS scores and ODI scores after surgery. OT was negatively correlated with the number of surgeries performed (*P* < 0.001, *r* =  −0.43). The learning curve of Mis-TLIF with BDUA was steep, with OT tapered to steady state in 43 cases.

**Conclusion:**

Compared with Open-TLIF with bilateral decompression, Mis-TLIF with BDUA can achieve equivalent clinical outcomes, lower PDV and LOS, and lower complication rates. Although this procedure took longer, it could be a viable alternative for the treatment of DLD after a steep learning curve.

## Introduction

Degenerative lumbar diseases (DLD) are very common in middle-aged and elderly people and requires surgical interventions to improve the related symptoms if conservative treatment fails [1, 2]. Lumbar decompression with fusion surgery such as Open Transforaminal Lumbar Interbody Fusion (Open-TLIF) is a classic surgical approach [3, 4]. For patients with bilateral lower extremity symptoms or preoperative contralateral foramen stenosis, bilateral decompression was usually recommended to avoid the risk of residual symptoms [5–7]. Minimally Invasive Transforaminal Lumbar Interbody Fusion (Mis-TLIF), which was first descried by Foley et al. [8] as a clinically equivalent and less invasive technique to Open-TLIF, is also widely adopted for the treatment of DLD [9–16]. Additionally, bilateral decompression via unilateral approach (BDUA), which was first descried by Spetzger et al. [17, 18]. is also believed to relieve bilateral lower extremity symptoms in DLD. Lin et al. firstly described the surgical procedure of Mis-TLIF combined with BDUA. He found that Mis-TLIF combined with BDUA was an effective and safe method for the treatment of bilateral foramen stenosis [19]. Our previous research also found that Mis-TLIF combined with BDUA can achieve good clinical outcomes in DLD patients [20]. However, there is no comparison study between Mis-TLIF combined with BDUA and Open-TLIF combined with bilateral decompression. It remains unknown whether the procedure of Mis-TLIF combined with BDUA can replace the procedure of Open-TLIF combined with bilateral decompression. Therefore, this study intends to explore the clinical outcomes of the two surgical procedures for DLD patients with bilateral lower limb symptoms.

## Methods

We retrospectively analyzed the prospectively collected clinical outcomes of consecutive patients with DLD in the two groups (Mis-TLIF combined with BDUA VS Open-TLIF combined with bilateral decompression) from January 2016 to January 2020. The conduct of this study was reviewed and approved by the Ethics Committee of Xinqiao Hospital.

### Inclusion criteria

(1) DLD including degenerative lumbar spondylolisthesis (DLS) and degenerative lumbar spinal stenosis (DLSS). (2). Patients who have underwent single-segment Open-TLIF combined with bilateral decompression or Mis-TLIF combined with BDUA. (3). Patients with more than 24 months follow-up.

### Exclusion criteria

(1) Patients who didn’t receive bilateral decompression. (2) Patients with lumbar spondylolisthesis greater than or equal to grade II (Meyerding classification). (3) Patients who have underwent single-segment Open-TLIF or Mis-TLIF due to other spinal disorders such as spinal fracture, tumor, infection or Degenerative spinal deformity etc. (4) Patients with ankylosing spondylitis or diffuse idiopathic skeletal hyperostosis disease. (5) Patients with serious chronic diseases that may interfere with clinical outcome such as Alzheimer's disease, Parkinson's disease, myocardial infarction, cerebral infarction, etc. (6) Patients with less than 24 months of follow-up.

### Clinical assessment

We retrieved basic patient information based on the hospital's registry system. The basic patient's information included the patient's age, gender, BMI (Body Mass Index), diagnosis, surgical procedure, operative time (OT), estimated blood loss (EBL), postoperative drainage volume (PDV), and length of stay (LOS). In addition, clinical assessments included the low back and leg Numerical Rating Scale (NRS) scores preoperatively, at discharge and 2 years postoperatively and Oswestry Disability Index (ODI) preoperatively and 2 years postoperatively. All patients finished the NRS scores and ODI questionnaires face-to-face with an interviewer preoperatively, at discharge and face-to-face with an interviewer or by telephone 2 years postoperatively.

### Radiological assessment

The degree of lumbar spondylolisthesis was evaluated according to Meyerding classification. Adjacent segment disease (ASD) is defined as a recurrence of symptoms in the low back or legs after primary surgery and is supported by corresponding radiological evidence. The radiological finding included significant disc degeneration, herniation, degenerative stenosis, segmental instability, spondylolisthesis and retrolisthesis at adjacent segment [21]. All radiological assessment was performed by a qualified radiologist.

### Surgical methods

#### Mis-TLIF combined with BDUA

**Fig. 1:**
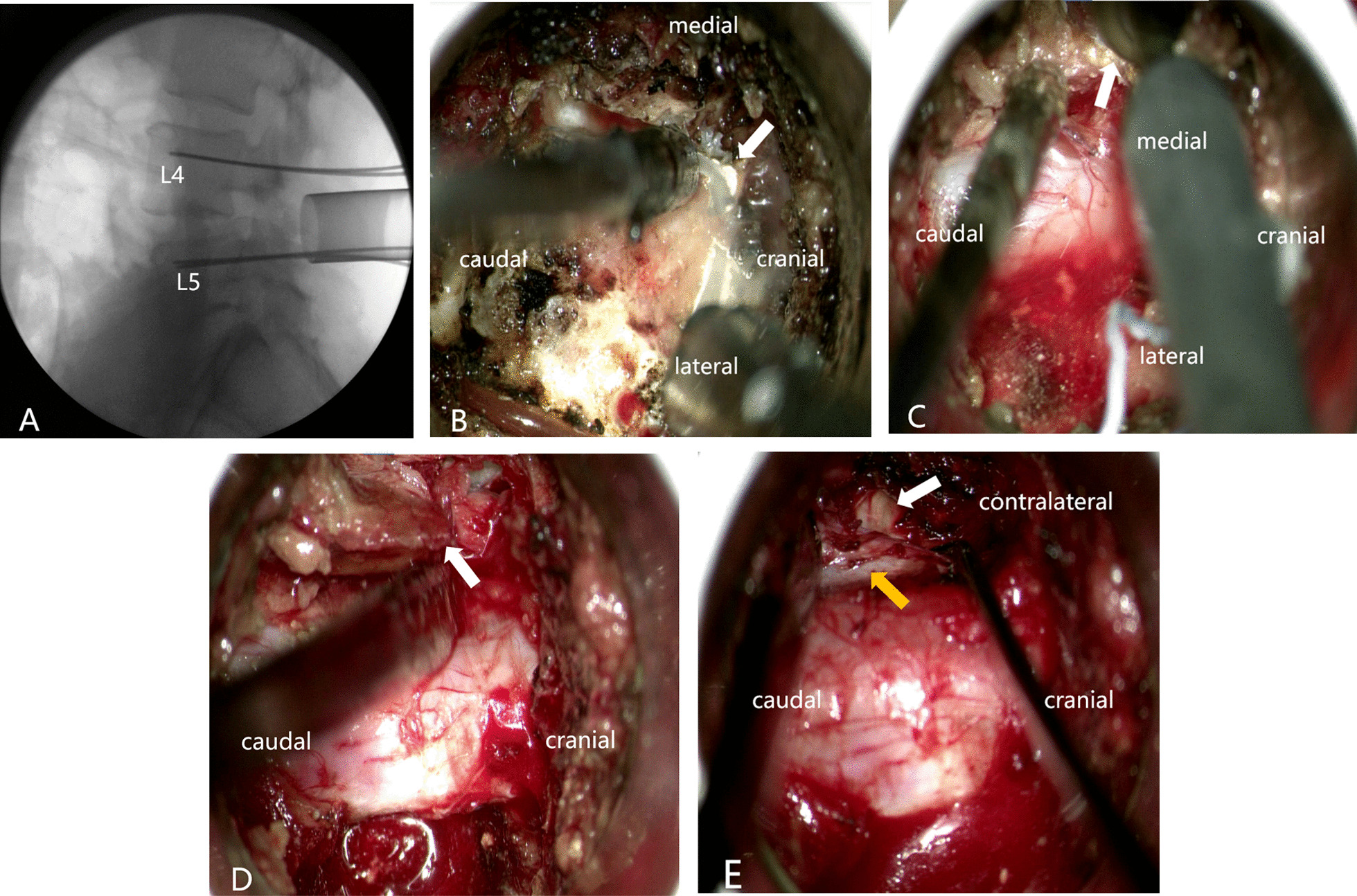
**A** The insertion of a guidewire prior to the insertion of a pedicle screw. **B** remove the articular process of L5 (white arrow). **C** grind off the root of bottom of spinous process (white arrow). **D** grind off the inner edge of contralateral L5 upper articular process and remove the ligamentum flavum (white arrow). **E** contralateral nerve root (yellow arrow) and intervertebral disc (white arrow)

The procedure of Mis-TLIF combined with BDUA was based on the methods we previously reported [22]. A case of bilateral decompression via left approach in L4/5 segment was selected to describe the surgical procedure. The patient was placed in prone position after general anesthesia. The L4/L5 interverteral disc space and bilateral pedicles of L4 and L5 vertebrae were marked on the skin by AP and lateral fluoroscopy, respectively. Incision along the surface projection of L4 and L5 pedicle was made bilaterally, and four guide wires were implanted into L4 and L5 pedicles through the multifidus and longissimus spaces under fluoroscopy. The first dilator was inserted through the left incision to touch the inferior edge of L4 lamina by splitting the multifidus muscle (Fig. 1A). After sequentially dilating, a tapered working tube with an entrance diameter of 26 mm and a base diameter of 22 mm (Zista, Bosscom Technology, Chongqing, China) [23] was docked on the left L4/5 facet joint between the L4 and L5 pedicle guide wires. Under the microscope (Carl Zeiss, Inc., Oberkochen, Germany), a 6 mm diamond bur was used to remove the overlapping osteophytes to expose the facet joint clearly. The facet joint including partial L4 lamina was totally resected using a 2 mm cutting-edge burr (Fig. 1B). After removing the ligamentum flavum and intraforanimal ligament, the left L5 nerve root and partial dura mater were exposed. Then the narrow lateral recess and intervertebral foramen were enlarged by Kerrison rongeur, and the nerve root was fully decompressed and pulled slightly to the middle line to expose the disc space, which was mainly located in the intervertebral foramen including partial canal region. Typical TLIF was performed as the open procedure. Autogenous and allogeneic bone graft were inserted for interbody fusion followed by implantation of an intervertebral cage with appropriate size.

Then, the working tube was tilted to the opposite side, and a 6 mm diamond bur was used to grind off the bottom of spinous process until the contralateral ventral lamina and inner part of L4 inferior articular process were removed (Fig. 1C). Finally, the inner edge of contralateral L5 upper articular process was grinded off, which was a feeling of compact cortical bone (Fig. 1D). After the contralateral ligamentum flavum was removed, the contralateral recess was enlarged, and the whole dura and contralateral L5 nerve root were totally exposed and released naturally due to remove of the dorsal “roof” structure (Fig. 1E). After definite hemostasis, a drainage tube was placed close to the decompression site and the working tube was removed. Pedicle screws were implanted through guide wires and titanium rods were inserted percutaneously, which was finally confirmed by fluoroscopy. The wound was then closed in layers in the usual manner.

Open-TLIF combined with bilateral decompression.

The posterior midline approach was used. In addition to the traditional TLIF procedure, contralateral articular process was partially removed and lateral recess was enlarged to release contralateral nerve roots.

### Statistical method

A Kolmogorov–Smirnov test was used to test the data distribution. The continuous variables (mean ± standard deviation) were examined by a t-test or Mann–Whitney U test. The categorical variables were examined by a Chi-square (fisher’s exact) test. A Spearman’s coefficient of rank correlation (rho) was used to assess the learning curve of Mis-TLIF combined with BDUA. A logarithmic curve was used to fit the relationship between the number of surgeries and OT. P value < 0.05 is considered as statistically significant difference between the two groups. Statistical analyses were performed by SPSS Statistics software (version 26.0, IBM Corp., Armonk, NY, USA).

## Results

### Demographics

**Fig. 2:**
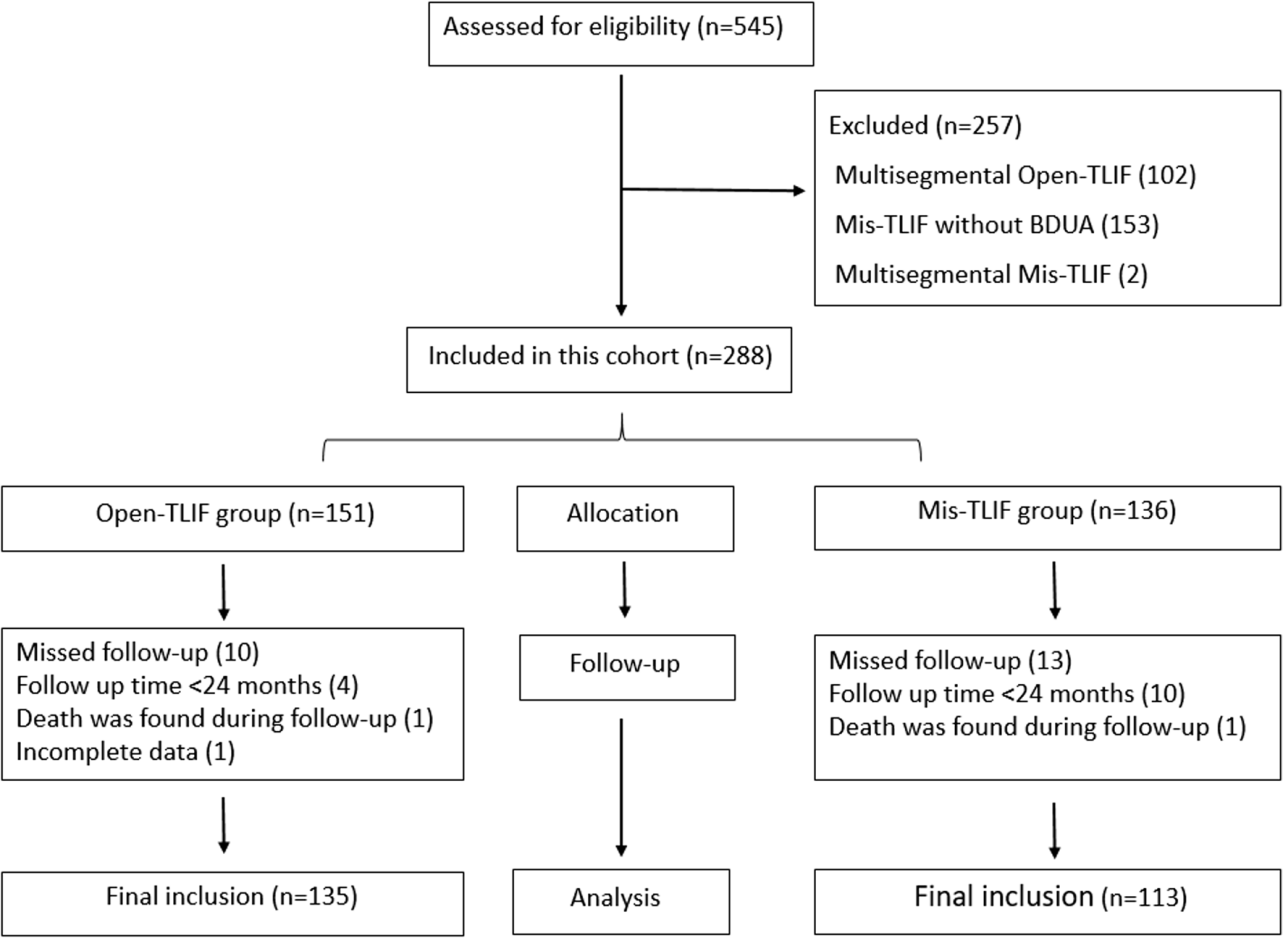
The inclusion and exclusion flowchart of this study

The inclusion and exclusion flowchart of this study is shown in Fig. 2. 545 patients who received TLIF procedures were enrolled initially. 135 cases of Open-TLIF combined with bilateral decompression and 113 cases of Mis-TLIF combined with BDUA were finally included. The demographics of the patients are shown in Table 1. There were 133 cases of DLS (grade I Meyerding Classification) and 115 cases of DLSS. In Open-TLIF group, there were 78 cases of DLS and 57 cases of DLSS. In Mis-TLIF group, there were 55 cases of DLS and 58 cases of DLSS. The mean age, BMI and follow-up time were 56.0 ± 11.3 years, 25.0 ± 3.1 kg/m^2^ and 56.9 ± 14.8 months, respectively. The mean age of Mis-TLIF group was significantly higher than that of Open-TLIF group (59.1 ± 10.0 vs 53.4 ± 11.9, *P* < 0.001).

**Table 1:** The demographics of Open-TLIF and Mis-TLIF groups

	Open-TLIF (n = 135)	Mis-TLIF (n = 113)	*P* value
Gender (men/women)	52/83	49/64	0.439
Age (years)	53.4 ± 11.9	59.1 ± 10.0	< 0.001
BMI (kg/m^2^)	24.7 ± 2.9	25.3 ± 3.3	0.131
Operative time (minutes)	154.7 ± 41.1	212.4 ± 56.2	< 0.001
Postoperative drainage (ml)	332.0 ± 178.5	126.1 ± 150.1	< 0.001
Length of stay (days)	7.3 ± 2.5	6.4 ± 2.0	0.002

### Clinical outcomes

The clinical outcomes are shown in Tables 2, 3 and 4. The mean OT of Mis-TLIF group was significantly higher than that of Open-TLIF group (212.4 ± 56.2 vs 154.7 ± 41.1, *P* < 0.001). There was no significant difference in intraoperative EBL between the two groups. The PDV and LOS of Open-TLIF group were significantly higher than those of Mis-TLIF group. There was no significant difference in low back, leg NRS scores between the two groups preoperatively, at discharge and 2 years postoperatively, respectively. There was no significant difference in ODI between the two groups preoperatively and 2 years postoperatively.

**Table 2:** Comparison of low back NRS scores of Open-TLIF and Mis-TLIF groups

Low back NRS score	Open-TLIF (n = 135)	Mis-TLIF (n = 113)	*P* value
Preoperative	3.9 ± 2.5	3.5 ± 2.3	0.198
Discharge	0.5 ± 0.8	0.7 ± 0.9	0.153
Postoperative 2 years	1.2 ± 1.5	1.1 ± 1.4	0.546
P value (Pre vs Dis)	< 0.001	< 0.001	
P value (Pre vs Po 2 y)	< 0.001	< 0.001

**Table 3:** Comparison of leg NRS scores of Open-TLIF and Mis-TLIF groups

Leg NRS score	Open-TLIF (n = 135)	Mis-TLIF (n = 113)	*P* value
Preoperative	4.4 ± 3.1	4.5 ± 2.6	0.724
Discharge	0.5 ± 1.1	0.6 ± 0.9	0.276
Postoperative 2 years	0.6 ± 1.1	0.7 ± 1.4	0.384
P value (Pre vs Dis)	< 0.001	< 0.001	
P value (Pre vs Po 2 y)	< 0.001	< 0.001

**Table 4:** Comparison of ODI of Open-TLIF and Mis-TLIF groups

ODI	Open-TLIF (n = 135)	Mis-TLIF (n = 113)	*P* value
Preoperative	47.9 ± 20.1	46.7 ± 16.9	0.603
Postoperative 2 years	13.5 ± 14.7	12.9 ± 13.4	0.733
P value (Pre vs Po 2 y)	< 0.001	< 0.001	

### Complications and treatments

Complications of the patients are shown in Table 5. The complication rate (20/135: 14.8% vs 7/113, 6.2%, P = 0.030) of Open-TLIF group were significantly higher than that of Mis-TLIF group. However, there was no significant difference in the reoperation rate (4/135: 3.0% vs 2/113, 1.8%, *P* = 0.691) and ASD rate (3/135: 2.2% vs 0/113, 0%, *P* = 0.253) between the two groups.

**Table 5:** Perioperative complications of Open-TLIF and Mis-TLIF groups

Complications	Open-TLIF (n = 135)	Mis-TLIF (n = 113)
Poor wound healing	3 (2 reoperation)	
Deep infection	1 (1 reoperation)	
Cerebrospinal fluid leak	8	2
Postoperative pyrexia	4	
Pulmonary infection		1
Epidural hematoma		2 (2 reoperation)
Nerve root compression	1 (1 reoperation)	
Adjacent segment disease	3	
Cage loosening and migration		1
Screw loosening		1

In Open-TLIF group, 3 cases had poor wound healing. Two cases were cured by debridement under local anesthesia and the other was by conservative treatment. One patient with deep infection was cured by debridement and antibiotic treatment. All 8 patients (5.9%) with cerebrospinal fluid (CSF) leakage were treated with delayed removal of drainage tube (about 10 days), and the drainage tube was removed when the incision was almost healed. By this method, poor wound healing occurred in only one case and was treated by debridement operation (count in poor wound healing). Intermittent fever occurred in 4 cases. One of them exceeded 39 ℃. No infection of incision or other sites was found in the 4 patients. All the 4 patients recovered after symptomatic treatment. One of the four patients with fever had a CSF leakage. One patient was diagnosed as postoperative ganglionitis because of aggravated postoperative lower extremity pain in the innervation area from the surgical segment. The patient’s symptoms were relieved after nerve root block at the surgical site. ASD was found in 3 patients according to postoperative symptoms and magnetic resonance imaging (MRI), none of them underwent reoperation.

In Mis-TLIF group, CSF leakage occurred in 2 patients (1.8%). All the drainage tubes were removed within 72 h postoperatively, after another 12 h bed rest, they were allowed to walk. The incisions of the 2 patients healed well. One patient developed pulmonary infection, which was cured by antibiotic treatment. Two patients had severe low back pain after operation, and one of them had symptoms of cauda equina injury and another patient had severe right lower extremity pain. The postoperative MRI suggested epidural hematoma. The patient with right lower extremity pain received timely revision treatment and had significant improvement of the pain. For another patient, even after timely debridement, there were still some residual symptoms of cauda equina injury in postoperative follow-up. One patient had cage loosening and migration, and one patient had screw loosening postoperatively. Neither patient underwent reoperation.

### Learning curve

**Fig. 3:**
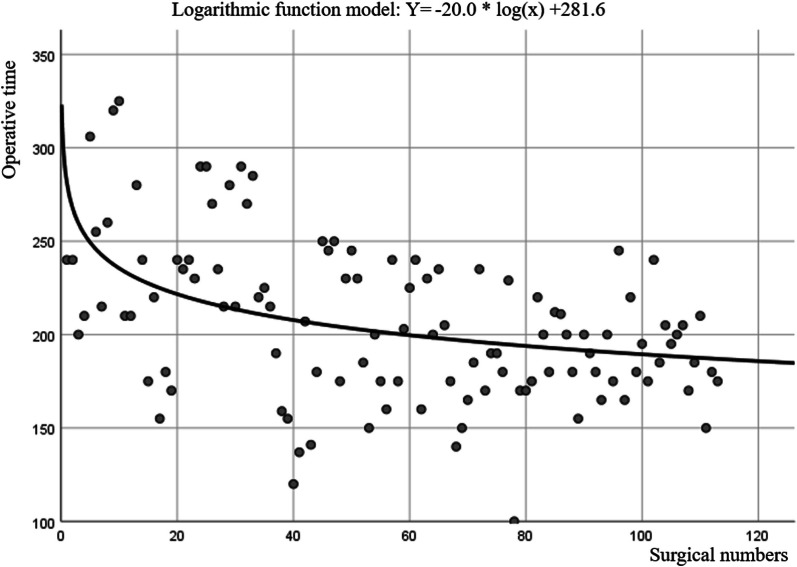
The relationship between operative time and surgical times

In Mis-TLIF group, the Spearman's coefficient analysis indicated that the OT was negatively correlated with the number of surgeries (*P* < 0.001, r = −0.43). The learning curve of Mis-TLIF combined with BDUA is shown in Fig. 3 (*P* < 0.001, Y = −20.0*log(x) + 281.6). Patients underwent Mis-TLIF combined with BDUA surgery experienced a gradual decrease of OT in the early stages, with OT tapered to steady state at the 43 cases. In addition, we found that the perioperative complication rate was higher in the former 43 patients than in the later 70 patients (6/43, 14.0% VS 1/70, 1.4%, *P* = 0.012).

## Discussion

We retrospectively analyzed the clinical outcomes of consecutive patients with DLD in the two groups. The OT was significantly higher in Mis-TLIF group, while the PDV and LOS were significantly higher in Open-TLIF group. Additionally, the complication rate was significantly higher in Open-TLIF group than in Mis-TLIF group. Finally, we found a steep learning curve for the procedure of Mis-TLIF combined with BDUA and stabilization of OT in the 43rd cases.

Compared with Open surgery, minimally invasive surgery has the advantage of avoiding direct dissection of paravertebral muscles [24]. This allows decompression or fusion surgery to be performed with minimal tissue damage. At present, there are some comparative studies on MIS-TLIF and Open-TLIF for DLS or DLSS [9–16]. Singh et al. found that OT, anesthesia time, Visual Analog Scale scores, EBL and LOS can be significantly reduced through Mis-TLIF compared with Open-TLIF [9]. A meta-analysis by Phan et al. also show that Mis-TLIF can reduce blood loss and infection rate compared with Open-TLIF [10]. More than one study has found that Mis-TLIF is superior to Open-TLIF in perioperative clinical outcomes, while no significant difference was found in the long-term outcomes between the two procedures [11–14]. However, a recent multicenter prospective study by Chan et al. found that Mis-TLIF is superior to Open-TLIF in long-term clinical outcomes for the treatment of grade I DLS [25]. Moreover, Mis-TLIF has been proposed as a viable alternative to Open-TLIF for treating DLD [11, 14]. However, whether Mis-TLIF combined with BDUA can replace Open-TLIF in patients requiring bilateral decompression remains unknown.

Cheng et al. compared the clinical and radiological outcomes of TLIF combined with BDUA and PLIF combined laminectomy for DLS. They found similar clinical outcomes of the two surgical procedures, and TLIF combined with BDUA appeared to be associated with less postoperative lumbar discomfort and faster recovery [26]. Huang et al. compared the clinical and radiological outcomes of TLIF via Wiltse approach combined with BDUA and Open-TLIF for lumbar degenerative disc disease. The authors found that BDUA can better protect the muscles on the opposite side from fatty infiltration [27]. In this study, we found shorter LOS and less PDV in Mis-TLIF group. This suggests that Mis-TLIF is superior to Open-TLIF in perioperative outcomes. The NRS scores and ODI of the two groups were significantly improved 2 years postoperatively. Furthermore, there was no significant difference of NRS score and ODI between the two groups preoperatively and postoperatively. This indicates that the pain and functional scores of the two procedures are equivalent.

**Fig. 4:**
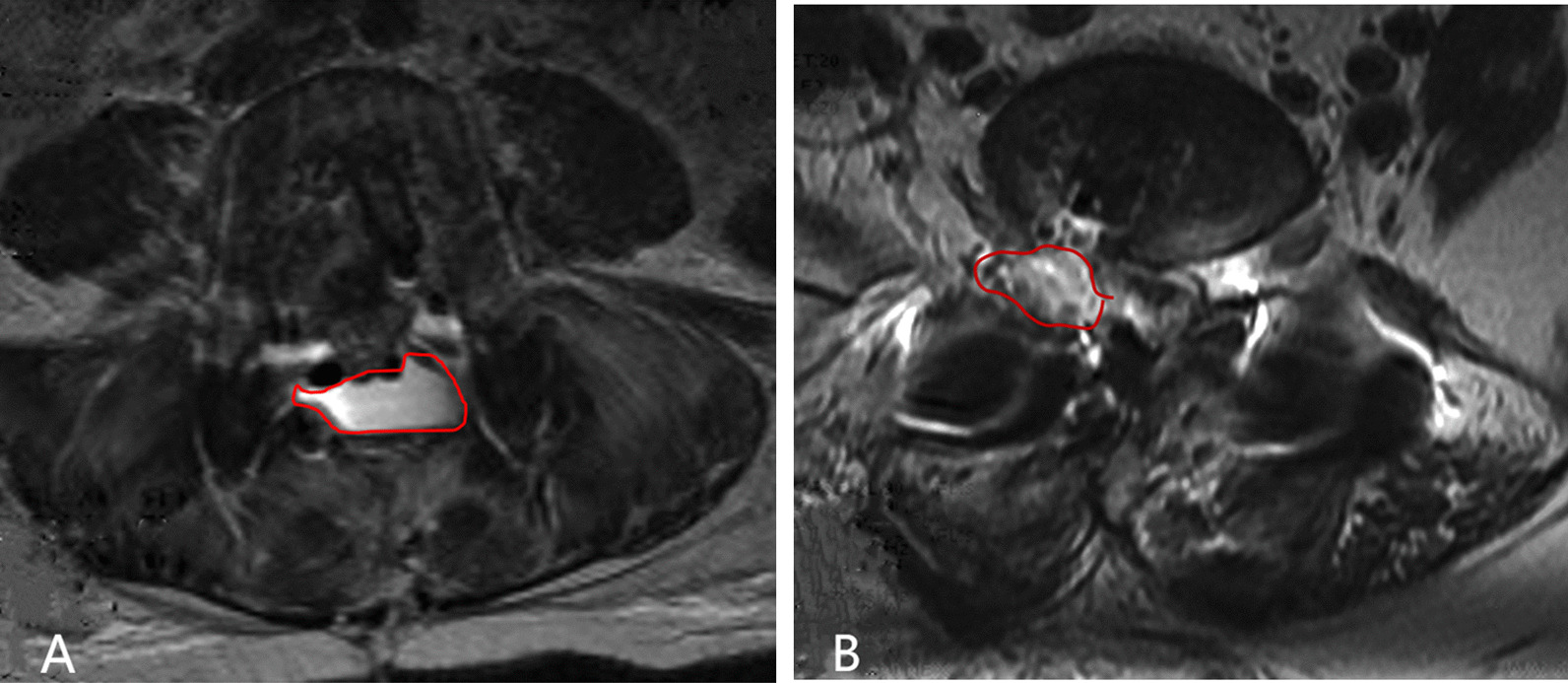
**A** the CSF leakage after Open-TLIF (in the red circle). **B** the CSF leakage was “restricted” in a narrow space after Mis-TLIF (in the red circle)

The difference of complication rate between Open-TLIF and Mis-TLIF is controversial [10, 12–15]. Hammad et al. found no significant difference in complication rate between Mis-TLIF and Open-TLIF [12], whereas most studies found a higher complication rate with Open-TLIF [10, 13–15]. In this study, we found that the overall complication rate was lower in Mis-TLIF group. CSF leakage due to dural tear was 5.9% (8/135) in Open-TLIF group and 1.8% (2/113) in Mis-TLIF group. One possible reason was that the Mis-TLIF procedure in this cohort was performed under a microscope, which allowed the surgeon to gain a clearer view of the surgical field. The management of CSF leakage was different between the Mis-TLIF group and Open-TLIF group. CSF spread under the lumbodorsal fascia due to extensive dissection of the multifidus muscle in Open-TLIF. If it is not drained out of the wound early after surgery, it will accumulate under the lumbodorsal fascia and increase the tension of the incision (Fig. 4A), thus affecting wound healing. Therefore, we extend the drainage time (about 10 days) to strive for wound healing. In the Mis-TLIF group, after removal of the working tube, the dilated multifidus muscle was naturally reset, thus greatly reducing the local wound space, and the leaked CSF was “restricted” in a narrow space without affecting the wound healing (Fig. 4B). Therefore, we usually remove the drain early after surgery (usually within 72 h). In addition, there were no patients with poor wound healing or infection in the Mis-TLIF group, whereas they occurred in four patients in Open-TLIF group. This also further reflects that MIS-TLIF has less trauma to the surgical site than Open-TLIF, thus affecting the healing of the incision.

**Fig. 5:**
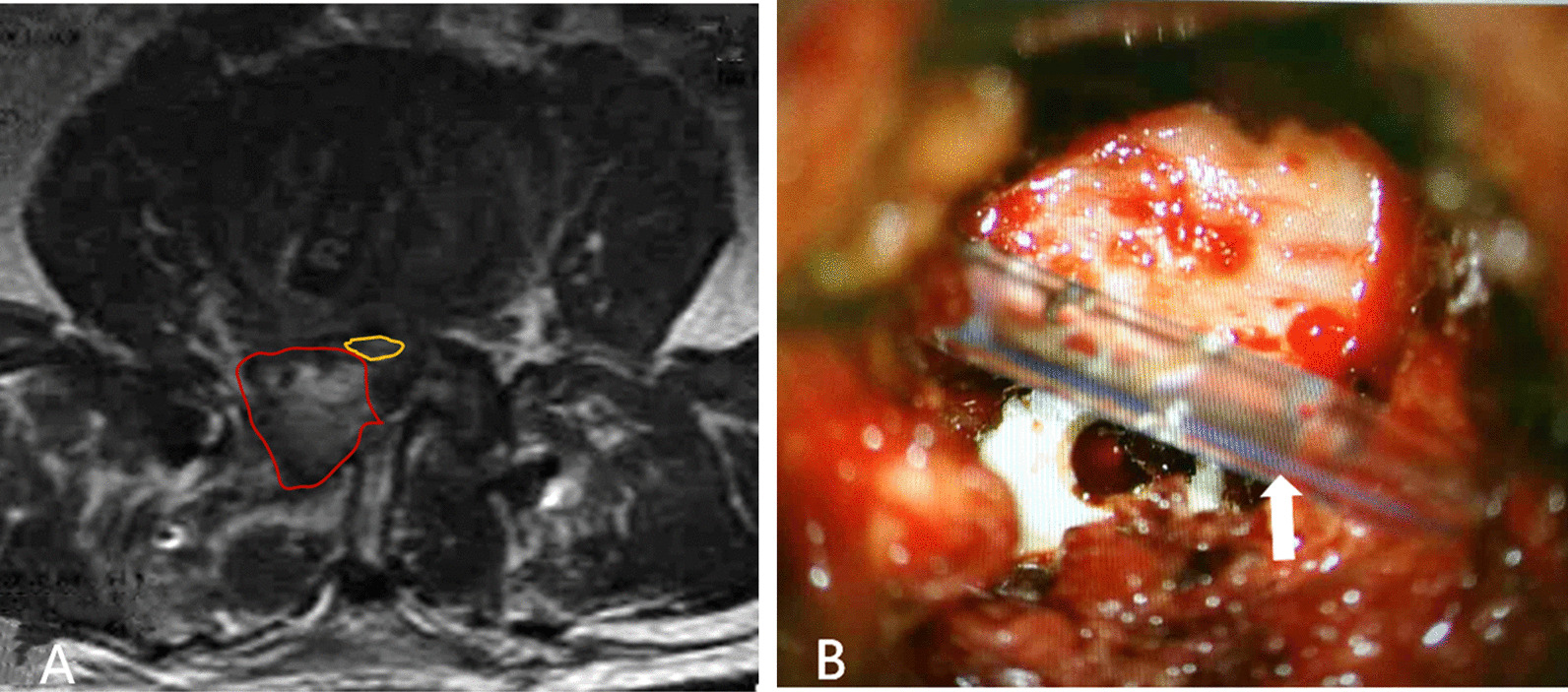
**A** epidural hematoma at operation area (in the red circle) and compressed nerve (in the yellow circle). **B** after removing the epidural hematoma, a drainage tube was placed close to the decompression site (white arrow)

It is important to note that in the Mis-TLIF group, two cases developed postoperative epidural hematoma (Fig. 5A). Although both patients underwent timely revision surgery, one patient had residual symptom of cauda equina injury. Actually, there was a potential risk of epidural hematoma in the BDUA procedure. On the one hand, the operating area (a 22 mm base diameter of working tube) was limited, which put forward a high requirement for hemostasis, otherwise a very small amount of bleeding might cause an epidural hematoma after removing the tube. On the other band, contralateral epidural bleeding is hard to control, especially in inexperienced hands. The limited space between the base of spinous process and the dura which was inflated after decompression restricted the smooth draining of the contralateral side. Therefore, adequate hemostasis, removal of any hemostatic material, which could block draining and proper drainage placement (Fig. 5B) are imperative when ending BDUA. This is the main way to prevent such complications.

A recent meta-analysis by Heemskerk et al. found there was no significant difference in reoperation rate (3.0% vs 2.4%) and ASD rate (12.6% vs 12.4%) between Mis-TLIF and Open-TLIF [11], while Mooney et al. found a lower reoperation rate in Mis-fusion surgery [28]. In this study, we did not find a significant difference in the reoperation rate (3.0% vs 1.8%, *P* = 0.691) between the two groups. However, 3 cases occurred ASD in Open-TLIF group while no case occurred ASD in Mis-TLIF group. The possible reason is that Mis-TLIF can minimize the iatrogenic injury of multifidus muscle by avoiding direct dissection. There was less multifidus atrophy after Mis-TLIF compared with Open-TLIF [23, 29, 30]. The study by Sun et al. found that the degree of disc degeneration and multifidus muscle atrophy were positively correlated [31]. Many studies have shown that the multifidus muscle is very important for maintaining the stability and sagittal alignment of the lumbar spine [32–34]. Therefore, compared with Open-TLIF, Mis-TLIF can better maintain the stability and sagittal alignment of the local lumbar spine and then reduce the risk of ASD by protecting against multifidus atrophy.

The learning curve of Mis-TLIF has been reported in previous studies [35–39]. A recent review by Ahn et al. found the mean cutoff point that surgeons were proficient in Mis-TLIF was about 31.33 ± 11.98 (range 13‒45) cases [35]. However, although the BDUA technique is widely used, there are no reports of the learning curve for the procedure of BDUA or Mis-TLIF combined with BDUA until now. In this study, we first analyzed the learning curve of Mis-TLIF combined with BDUA. We found that the OT was negatively correlated with the number of surgeries. The learning curve for this technique is steep. The OT of the cases leveled off after the 43rd operation. Additionally, the complication rate of the former 43 cases (6/43, 14.0%) is higher than that of the latter 70 cases (1/70, 1.4%), which can further prove that the 43rd case is the cutoff value of Mis-TLIF combined with BDUA.

## Limitations

The retrospective nature of this study leads to unavoidable basis. For example, there was a significant difference in the age of the two groups of patients. In addition, this study did not investigate the postoperative computer tomography of all the patients. It is difficult to accurately evaluate the fusion rate of the two groups of patients. Finally, this study lacks clinical results in the short to medium term for patients, such as VAS scores and ODI at 3 months, 6 months, and 1 year after surgery.

## Conclusion

Compared with Open-TLIF with bilateral decompression, Mis-TLIF with BDUA can achieve equivalent clinical outcomes, less PDV, LOS and lower complication rate. Even though this procedure took longer, Mis-TLIF with BDUA can be a viable alternative of Open-TLIF with bilateral decompression for the treatment of DLD after a steep learning curve.

## Data Availability

The data is available under reasonable request (Bo Huang).

## Abbreviations

ASD: Adjacent segment disease
BMI: Body mass index
BDUA: Bilateral decompression via unilateral approach
CSF: Cerebrospinal fluid
DLSS: Degenerative lumbar spinal stenosis
DLD: Degenerative lumbar diseases
EBL: Estimated blood loss
LOS: Length of stay
Mis-TLIF: Minimally invasive transforaminal lumbar interbody fusion
MRI: Magnetic resonance imaging
NRS: Numerical Rating Scale
ODI: Oswestry disability index
OT: Operative time
PDV: Postoperative drainage volume
